# A compositional approach towards understanding recovery sleep dynamics

**DOI:** 10.1093/sleep/zsag242

**Published:** 2026-09-02

**Authors:** David Negelspach, Alisa Huskey, Kathryn E R Kennedy, Jungwon Cha, Daniel B Forger, William D S Killgore

**Affiliations:** Social, Cognitive, and Affective Neuroscience Lab, Department of Psychiatry, College of Medicine, University of Arizona, Tucson, AZ, United States; Social, Cognitive, and Affective Neuroscience Lab, Department of Psychiatry, College of Medicine, University of Arizona, Tucson, AZ, United States; Social, Cognitive, and Affective Neuroscience Lab, Department of Psychiatry, College of Medicine, University of Arizona, Tucson, AZ, United States; Social, Cognitive, and Affective Neuroscience Lab, Department of Psychiatry, College of Medicine, University of Arizona, Tucson, AZ, United States; Department of Mathematics, University of Michigan, Ann Arbor, MI, United States; Social, Cognitive, and Affective Neuroscience Lab, Department of Psychiatry, College of Medicine, University of Arizona, Tucson, AZ, United States

**Keywords:** sleep architecture, sleep deprivation, recovery sleep, cognitive performance, psychomotor vigilance, mathematical modeling

Sleep restores cognitive functioning in the brain, but the specific parameters that constitute an “optimal” night of sleep remain up for debate. Sleep duration requirements vary from person-to-person and also vary from night-to-night [1], shifting what “optimal” means for any given episode. While it is well established that sleep duration strongly influences cognitive functioning, the sequence and composition of sleep stages represent core substrates of neurophysiological processes that contribute to cognitive recovery. Interdependence between sleep stages may lead to synergistic outcomes with respect to next-day functioning depending on an optimal balance.

Sleep is typically classified as either rapid eye-movement (REM) or one of three non-REM (NREM) stages. Throughout the night, these stages progress in cycles that contain the same general sequence but with varying durations, indicating a hierarchical structure and dynamic functional architecture. This pattern is shaped by both circadian and homeostatic processes [2], as well as by interdependence between sleep stages themselves. For example, initial slow-wave activity dissipates homeostatic pressure [3], thereby changing the subsequent drive for slow wave activity [4]. In contrast, REM sleep propensity is strongly modulated by circadian phase, with longer and more frequent REM episodes occurring closer to the circadian nadir [5]. Consequently, preceding stage durations may have downstream effects that affect the timing and transitions of subsequent cycles, shifting the overall composition across sleep.

Interactions between REM and NREM episodes also occur. NREM sleep influences the timing and likelihood of subsequent REM episodes [6]. Rodent models also show that REM sleep in turn reorganizes neural activity patterns that emerge during subsequent NREM periods, contributing to the renormalization of hippocampal excitability [7]. Such findings are consistent with models proposing a reciprocal and interdependent relationship between NREM and REM-generating systems [8]. Together, this supports the view that sleep architecture reflects an iterative process in which circadian, homeostatic, and stage-dependent mechanisms jointly regulate the temporal organization and functional dynamics of sleep. As such, analytical approaches that consider isolated durations of sleep stages without considering relative proportions may overlook effects associated with the interdependent balance across sleep architecture.

Evidence of the interdependence between sleep stages raise the possibility of emergent effects on next-day cognitive functioning. As polysomnographic data is inherently compositional, multivariate modeling requires considerations for spurious correlations introduced between stages [9]. This insight has sparked a recent shift to compositional data analysis approaches [10, 11]. For the first time, we apply this framework to recovery sleep following sleep deprivation to determine if the relative distribution between stages explains individual variability in next-day psychomotor responses.

## Methods

A detailed description of the experimental protocol can be found in [12]. Briefly, participants (n_male_ = 11, n_female_ = 9) underwent 39 hours of sleep deprivation with regular neurocognitive testing (including psychomotor vigilance) every six hours. Psychomotor vigilance tests (PVT) were administered digitally to measure reaction time by pressing a button in response to a timer that randomly appears on the screen. During free time, participants engaged in activities of their choice as long as it did not include physical exercise or lying down. Wakefulness was confirmed via closed-circuit cameras. Following deprivation, participants had a 9-hour recovery sleep with polysomnography. The study protocol was reviewed and approved by the University of Arizona Institutional Review Board. All participants provided informed consent prior to research activities. One participant was excluded as an outlier.

### Modeling

Sleep stages are a part of a fixed total duration, meaning a change in a single stage can affect the duration of another, potentially resulting in spurious correlations. Aitchison geometry together with isometric log ratios (ILR) resolves this issue by transforming interrelated variables into orthogonal components [13, 14]. As a result, multiple linear regression models more accurately represent the balance between stages while addressing the fixed sum constraint.


$$ {ILR}_i=\sqrt{\frac{i}{i+1}}\ln \left[\frac{x_{i+1}}{g{\left({x}_1,\dots, {x}_i\right)}^{1/i}}\right] $$

Isometric log ratios were encoded in a specified order to represent the balance between REM and NREM stages. ILR1 represents the balance between N1 (i = 1) and REM, ILR2 represents the balance between N2 (i = 2) relative to N1 and REM, and ILR3 represents the balance between N3 (i = 3) relative to N2, N1, and REM. The aforementioned ILR definitions were created to contrast REM with NREM stages, reflecting the traditional classification of sleep stages, and to specifically compare N3 relative to the remaining sleep architecture. Linear regression was applied to ILR transformed data to predict within-subject differences in PVT reaction time after recovery sleep while controlling for duration. Psychomotor vigilance recovery is defined as the difference in mean reaction time between sleep deprivation and post-recovery sleep, with higher values indicating greater improvement in response speed.

## Results

First, to illustrate the basic link between sleep duration and PVT performance, a bivariate regression model was fit to the data (Figure 1a; b_tst_ = .22, *p* = .0575). The compositional transform of sleep-stage data is shown in Figure 1b. Compositional regression modeling yielded significant predictors of ILR components 1 and 3, and total sleep time as expected (b_ilr1_ = -19.81, *p* = .013; b_ilr3_ = -33.01, *p* = .0085; b_TST_ = .24, *p* = .02; p_overall = .0067, R^2^ = .50). Results indicate that a greater relative contribution of N1 compared with REM was associated with less improvement in PVT reaction time (ILR1). Likewise, a greater relative contribution of N3 relative to REM, N1, and N2 was associated with a smaller within-subject performance improvement on the PVT (ILR3). By itself, N3 sleep was not significantly associated with PVT recovery (b = -.53, *p* = .35). The added variable plot (Figure 1c) illustrates the association between ILR3 and next-day reaction time after removing shared variance with other predictors. When compared to a sleep duration-only model, likelihood ratio test showed that adding ILR components significantly improved model fit (F(3, 14) = 5.06, *p* = .014), indicating that sleep composition provided explanatory value beyond total sleep time. A standardized coefficient plot showing the effect sizes of predictors is shown in Figure 1d.

**Figure 1:**
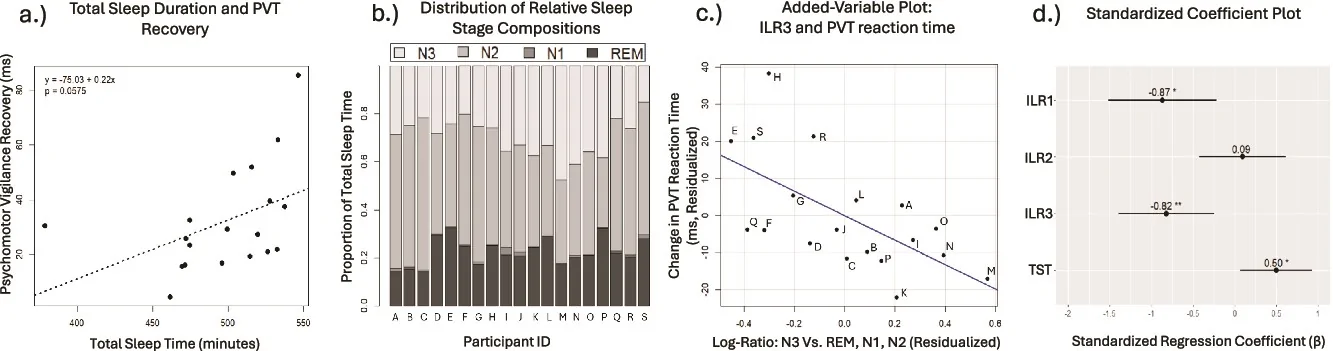
Sleep-Stage Compositions and Partial Regression of Psychomotor Recovery. Panel a shows the association between total sleep time and psychomotor vigilance recovery. The dotted line represents the fitted linear regression line, and the regression model equation/statistic are shown in the top left. Panel b shows individual sleep-stage compositions during recovery sleep for each participant. Panel c shows the isolated association between the ILR3 component and change in reaction time following recovery sleep. The solid line represents the association between ILR3 and change in reaction time after accounting for other ILR components and total sleep time (TST). Panel d shows the standardized coefficient plot illustrating the relative predictive strength of each variable along with their 95% confidence intervals.

## Discussion

In this study, we investigated how the temporal composition of recovery sleep architecture following 39 hours of deprivation is associated with improvements in psychomotor vigilance. Results confirm that individual differences in the relative distribution of time spent in each sleep stage significantly predicted the degree of improvement in next-day reaction time. Accounting for the combined composition of sleep stages significantly improved accounted variance in performance recovery as compared to sleep duration alone. This indicates that compositional balances between NREM and REM sleep may capture aspects of recovery sleep independent of the total amount of sleep obtained.

For a given sleep episode, the relative balance of individual sleep stages can differ significantly. Compositional variables encode inter-variable dependencies well-suited to capture emergent effects of relative sleep-stage balance that are not captured by individual stage durations. As opposed to fixed monotonic associations given by single duration-based variables, these ILRs capture multicomponent relationships among REM and NREM stages, allowing us to test whether specific patterns of sleep-stage distribution meaningfully predict cognitive outcomes. This is particularly relevant in biological systems such as sleep, where neurophysiological processes such as synaptic remodeling occurs in a complementary manner across sleep stages [15]. Unique and sequential aspects of synaptic remodeling occur in N2 [16], N3 [17–19], and REM [20, 21] that are complementary, suggesting that the relative balance between them has a consequential impact on synaptic homeostasis and, by extension, dynamics of neural communication. The utility of this approach therefore lies in specifically testing whether relative balances of sleep-stage distributions predict outcomes such as cognitive restoration that extend beyond single-stage durations.

Controlling for sleep duration, the complete model revealed two compositionally relevant ILRs associated with recovery of psychomotor vigilance following sleep deprivation. ILR1, which represents a greater dominance of N1 relative to REM, is negatively associated with psychomotor vigilance recovery. This result is consistent with previous sleep research, as N1 is typically considered non-restorative [22], relative to REM which is implicated in memory consolidation, emotional processing, and synaptic plasticity [23]. Greater N1 dominance relative to REM could reflect greater sleep fragmentation or difficulty progressing into deeper stages of sleep, both of which would be less conducive for restorative sleep.

Interestingly, modeling also revealed that a greater proportion of N3 sleep, compared to other stages, was negatively associated with next-day PVT performance. This was unexpected, as N3 sleep is commonly considered highly restorative. It is possible this result is indicative of individual differences in resilience to sleep loss, and thus recovery need [24]: Potentially, a dominant N3 rebound is indicative of excessive sleep debt which requires a more extensive recovery, and thus next-day performance is still inhibited. However, follow-up analysis reveals that N3 duration by itself did not predict next-day recovery. Alternatively, this result suggests that increases in N3 may not be invariably beneficial. Instead, N3 rebound following sleep deprivation may be most beneficial when accompanied by a corresponding compositional change in N2/N1/REM, as defined by the ILR3 component.

In summary, compositional analyses such as this provide a pathway to assess whether relative distributions between sleep stages convey aspects of recovery sleep that, in fact, can’t be captured with duration-based models. To be clear, this is not to downplay the importance of total sleep duration, as this is a monumentally important factor with a well-established history of consequential impact on cognition and health [25]. Rather, compositional analyses can reveal whether recovery is partially dependent on achieving a particular balance among sleep stages, consistent with the idea that the neurophysiological processes supported by each stage can be synergistic if balanced accordingly. As such, it’s possible that a fixed total sleep duration can yield different recovery outcomes depending on how that time is allocated across the sleep cycle. Here, we show that greater N1/REM and greater relative representation of N3 within the remaining sleep architecture are relevant compositions that are negatively associated with psychomotor vigilance recovery. Importantly, these associations concern a relative balance among multiple sleep stages rather than fixed monotonic association. Thus, these results encompass multiple stages interactions within an ILR that have a convergent effect on the composition, as opposed to a fixed monotonic relationship of specific stages.

Finally, the present findings are derived from a single night of recovery sleep in a relatively small sample size and should therefore be interpreted with caution. Future research should consider recovery sleep across multiple nights in a larger sample for further validation.
